# Closed Intratendinous Rupture of the Flexor Digitorum Profundus: A Rare Cause of Pediatric Trigger Finger

**DOI:** 10.7759/cureus.90819

**Published:** 2025-08-23

**Authors:** Pouya Mafi, Nagarjun N Konda, Matthew Venus

**Affiliations:** 1 Plastic Surgery, University Hospitals Coventry and Warwickshire NHS Trust, Coventry, GBR

**Keywords:** diagnostic uncertainty, finger trauma, pediatric hand surgery, plastic and reconstructive surgery, trigger finger disorder

## Abstract

Paediatric trigger finger (PTF) is a rare condition, far less common than paediatric trigger thumb, and often associated with anatomical anomalies or systemic disease. Unlike trigger thumb, which may resolve spontaneously, PTF usually requires surgical treatment. Trauma is an uncommon cause, and closed intratendinous rupture of the flexor digitorum profundus (FDP) has not previously been reported in a child following direct injury.

We present the case of a healthy 12-year-old boy who developed triggering of the right middle finger eight weeks after blunt trauma during rugby. Examination revealed a palpable nodule at the A2 pulley and paradoxical distal interphalangeal (DIP) joint extension during fist-making (lumbrical plus phenomenon). Ultrasound and MRI demonstrated intact FDP and flexor digitorum superficialis (FDS) tendons without an obvious lesion. Ongoing symptoms led to surgical exploration, which revealed a partial intratendinous FDP rupture with a scar nodule beneath an intact A2 pulley. Management included partial A2 pulley release, nodule excision, FDP repair with 5/0 polypropylene suture, and lumbrical release.

Early active mobilisation began within days postoperatively. At one year, the patient had a full, pain-free range of motion, normal grip strength, and no recurrence.

This is the first reported paediatric case of PTF caused by a closed, trauma-induced partial FDP rupture. The intratendinous tear produced a scar mass that impinged beneath the A2 pulley, mimicking more typical A1 pulley pathology. The lumbrical plus sign, rarely described in children and usually linked to complete ruptures, here resulted from a partial tendon injury. Standard imaging did not identify the lesion, highlighting the limitations of ultrasound and MRI in subtle intratendinous injuries. Careful clinical examination, particularly eliciting paradoxical extension, was critical to diagnosis.

Literature review indicates most PTF cases are idiopathic or related to systemic disease, with only ~5% linked to trauma. Surgical release yields higher resolution rates than conservative management (87% vs. 58%). Given the mechanical nature and delayed presentation of this lesion, surgery was both diagnostic and therapeutic. A comprehensive approach, pulley release, tendon repair, and lumbrical release, produced an excellent outcome.

PTF after blunt trauma can arise from closed intratendinous FDP rupture, even when imaging is unremarkable. The lumbrical plus sign is a valuable but under-recognised diagnostic clue. Early surgical exploration should be considered when suspicion remains high, as timely intervention can restore full function and prevent long-term disability.

## Introduction

Trigger finger (stenosing flexor tenosynovitis) is a condition in which a flexor tendon becomes transiently caught at a pulley, causing the digit to lock in flexion or extend with a painful snap. In adults, it is common (approximately 3% prevalence, higher in diabetics) and usually involves the A1 pulley [1]. In children, however, trigger digits are rare (estimated incidence <0.05%) and present differently [2]. Paediatric trigger thumb (flexion contracture due to a Notta’s nodule on the flexor pollicis longus) is about 10 times more common than paediatric trigger finger (PTF) [3]. Unlike trigger thumbs, which are often congenital or early acquired and may resolve spontaneously or with splinting, PTF is usually acquired later in childhood and almost always requires surgical intervention [4]. PTF can be associated with anatomical anomalies or systemic conditions (e.g., aberrant flexor digitorum superficialis (FDS) slips, mucopolysaccharidosis, juvenile idiopathic arthritis) and rarely resolves without treatment.

PTF remains a distinctly uncommon entity. A systematic review by Wong et al. found it occurs up to ten times less often than paediatric trigger thumb [5]. Most reported cases are idiopathic or linked to systemic disease. Trauma is a particularly rare cause: only about 5% of reported PTF cases follow trauma, usually in older children (≥7 years). We report a unique case of trauma-induced PTF caused by a closed intra-tendinous rupture of the flexor digitorum profundus (FDP) tendon. To our knowledge, this is the first case associated with direct trauma; only one prior case without trauma has been reported in a child. This report highlights key differences in presentation and management between PTF and the more common paediatric trigger thumb, reviews the relevant anatomy of the pulley system and lumbrical mechanism, and outlines diagnostic challenges. Imaging (ultrasound or MRI) may be useful to exclude structural lesions or assess tendon integrity [6]. However, as this case illustrates, standard imaging may fail to detect subtle intra-tendinous injury, and surgical exploration may be required when clinical suspicion is high.

## Case presentation

**Figure 1:**
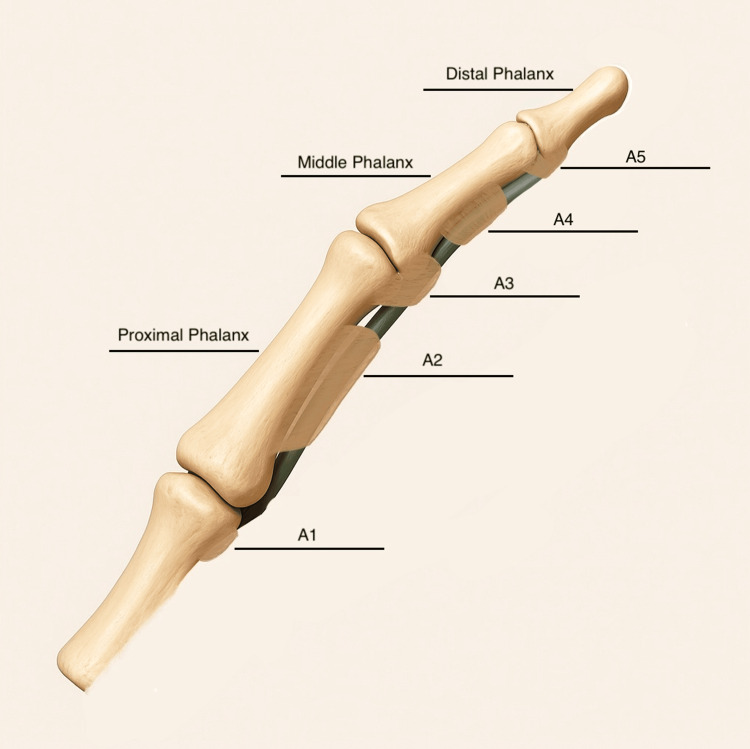
Schematic representation of the flexor tendon pulley system in a finger, showing the annular pulleys (A1-A5) in relation to the phalanges (proximal, middle, distal) and joints. The A1 pulley at the MCP joint is a common site of triggering in adults. The A2 and A4 pulleys (over the proximal and middle phalanx) are crucial to maintain the tendons close to the bone and prevent bowstringing during flexion. The A3 and A5 pulleys overlie the PIP and DIP joints, respectively, and are more compliant. MCP: metacarpophalangeal, PIP: proximal interphalangeal, DIP: distal interphalangeal. Image Credit: Authors' creation.

The flexor tendons are stabilised by annular pulleys (A1-A5) and cruciate pulleys (C1-C3), which form a fibro-osseous tunnel in each digit. The A1, A3, and A5 pulleys arise from the volar plates at the MCP, PIP, and DIP joints, respectively, while the A2 and A4 pulleys, attached to the proximal and middle phalanges, are critical for preventing bowstringing (Figure 1). In adults, pathological thickening of the A1 pulley or tendon nodules typically causes entrapment. In children, PTF may result from a mismatch between tendon size and pulley diameter, sometimes due to nodular or tendinous enlargement.

History

A 12-year-old boy presented to our hand clinic with triggering of the right middle finger after a blunt injury. Eight weeks earlier, he had been accidentally stamped on the hand during a rugby game. He experienced pain and swelling initially, which resolved within a week, but soon developed catching and locking of the middle finger on full flexion. The triggering persisted and gradually worsened, leading to referral. There was no open wound or fracture at the time of injury, and the patient had no prior finger problems. He was otherwise healthy, with no history of inflammatory or metabolic disorders.

Examination

On inspection, there was no obvious deformity or skin change, and the hand cascade appeared normal at rest. Palpation revealed a small, soft, mildly tender lump (~5 mm) on the volar aspect of the middle finger proximal phalanx, at the level of the A2 pulley. When attempting a full fist, the distal interphalangeal joint (DIPJ) of the middle finger paradoxically extended instead of flexing, producing a lumbrical plus deformity. With slower, careful flexion, the finger would 'trigger', as the tendon caught and then released, allowing the DIPJ to flex with a snap. Passive range of motion of the affected finger was full, including complete DIP flexion, confirming the joint itself was not stiff. Active range of motion was otherwise full in all joints of all fingers. Both FDP and FDS functions were intact in the affected finger, although FDP action at the DIPJ was limited as described. The finger could be fully extended actively and passively, with no fixed flexion contracture. The Bunnell test was normal, indicating no intrinsic tightness. Neurovascular examination was unremarkable.

Investigations

Given the traumatic onset and unusual examination findings, imaging was performed. Ultrasound of the middle finger flexor region demonstrated both FDS and FDP tendons in apparent continuity, gliding normally with passive motion. No discrete rupture was seen, although the FDP showed some thickening. Passive DIP flexion was full under dynamic ultrasound, but active DIP flexion remained limited. Based on these findings, a provisional diagnosis of stenosing tenosynovitis or a pulley injury (such as A2 strain or partial tear) was considered. MRI was then obtained to further assess the pulleys and exclude occult tendon pathology. The study showed intact flexor tendons without discontinuity or bowstringing, and the A2 pulley appeared preserved. There was no marrow oedema, tenosynovitis, or soft-tissue mass. The only subtle abnormality was a minor irregularity in the FDP, which was not definitively diagnostic. In summary, imaging failed to reveal a clear structural cause for the triggering. However, given the persistent symptoms and the paradoxical extension sign (highly suggestive of an FDP lesion), exploratory surgery was undertaken for definitive diagnosis and treatment.

Intraoperative findings

**Figure 2:**
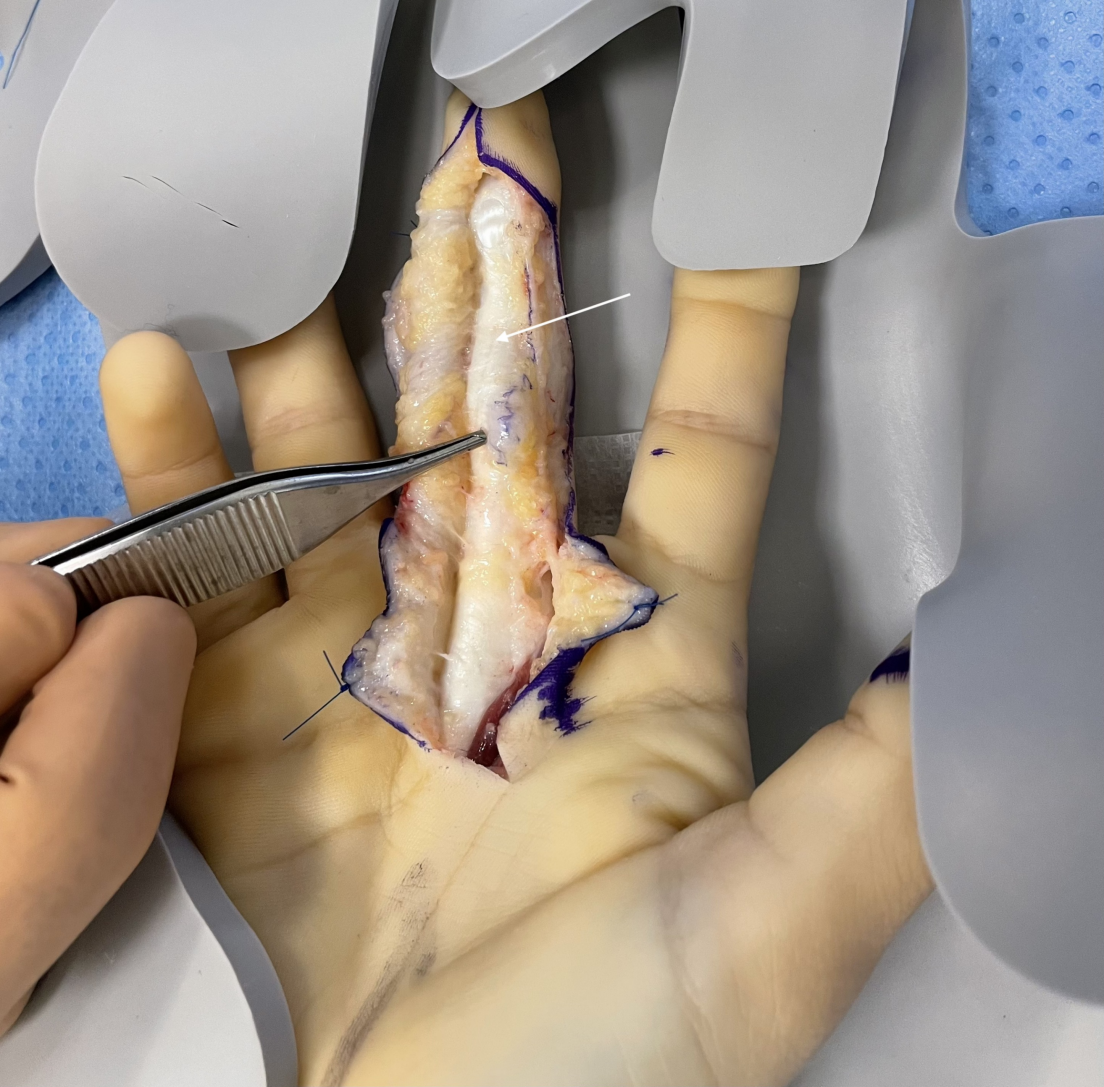
Intraoperative photograph demonstrating the FDP tendon and the site of the intratendinous rupture (tip of the forceps). The flexor tendon (white arrow) can be seen under the partially lifted A2. A bulging nodule of scar (scarred tendon tissue) is evident on the tendon’s radial aspect where the partial tear occurred. This confirmed that the triggering was caused by the torn tendon fibres impinging on the pulley.

Exploration of the flexor tendon sheath of the middle finger was performed under general anaesthesia and tourniquet control. A Bruner incision was made over the volar aspect of the proximal phalanx. On opening the sheath, a closed intratendinous rupture of the FDP tendon was identified beneath an intact A2 pulley (Figure 2). Specifically, there was a longitudinal split and partial tear involving the radial half of the tendon at the mid-proximal phalanx. The torn segment had retracted slightly, with fibrous tissue forming a spindle-shaped intratendinous nodule. The sheath itself was intact, confirming the closed nature of the injury. The A2 pulley was preserved but constrictive, with the fibrous nodule catching on its distal margin during flexion, accounting for the triggering.

**Figure 3:**
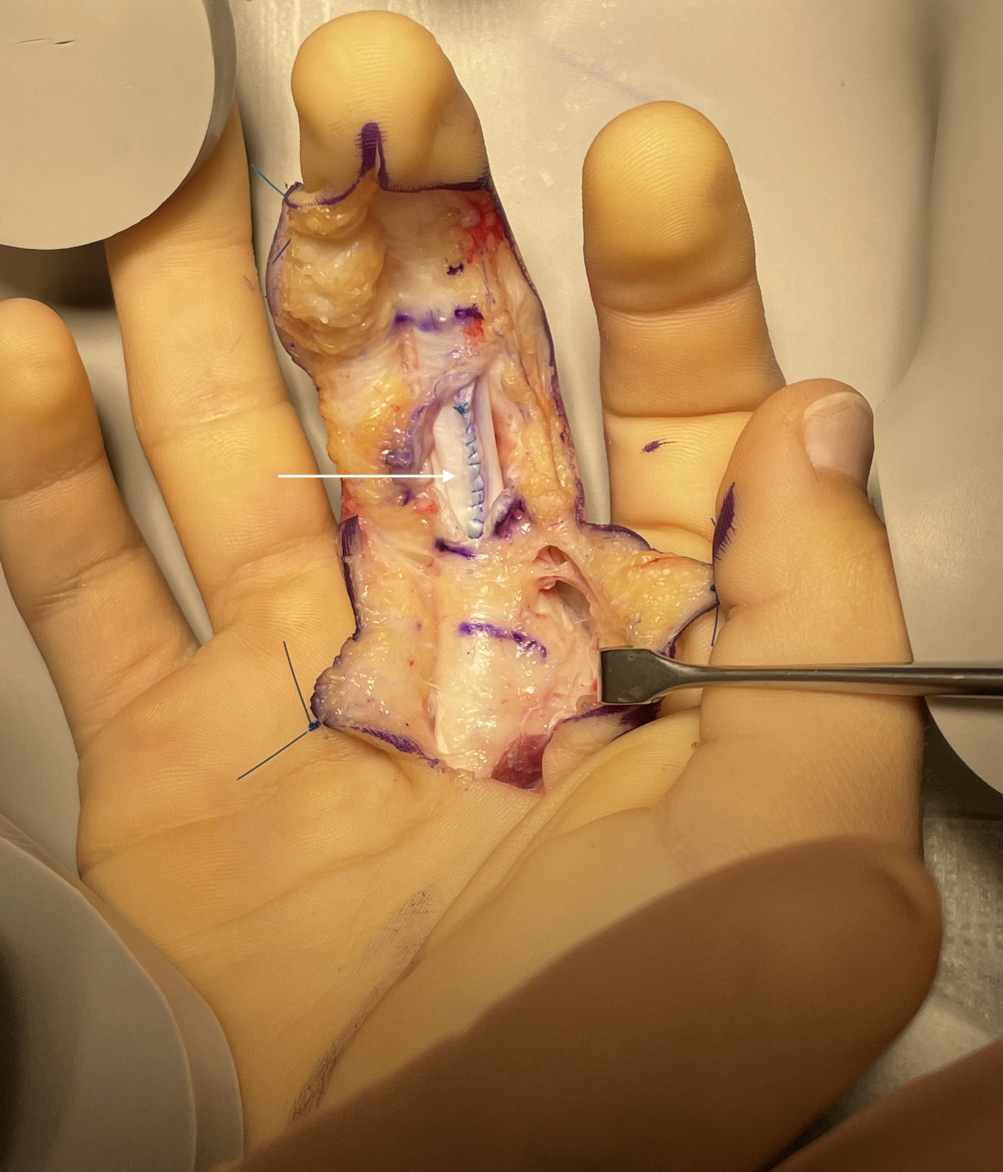
FDP tendon after repairing the intratendinous tear. The white arrow indicates the suture line closing the defect in the tendon. FDP: flexor digitorum profundus.

To restore smooth tendon gliding, a limited venting (partial release) of the A2 pulley was performed, followed by excision of the fibrous intratendinous nodule. The FDP tendon defect was then addressed: the tear margins were debrided, frayed fibres were trimmed, and the defect was repaired using interrupted 5-0 polypropylene (Prolene) sutures (Figure 3).

After the repair, the tendon was tested through a full range of motion; the bulk of the tendon now passed smoothly under the A2 pulley without catching. Intraoperative flexion-extension testing confirmed that triggering had been resolved by the combination of pulley release and tendon repair. We noted that the lumbrical muscle to the middle finger (which originates from the FDP tendon in the palm) appeared tight with the finger extended. The lumbrical was accessed through the same incision by tracing proximally and subsequently released. The incision was closed with absorbable sutures, and a light dressing was applied. Importantly, no postoperative splint was used, and the finger was not immobilised to encourage tendon glide and prevent adhesions.

Postoperative course

The patient commenced supervised hand therapy within a few days postoperatively. Early active motion was encouraged under the guidance of a certified hand therapist, without rigid splintage, given the partial nature of the tendon injury and secure repair. Rehabilitation focused on controlled range-of-motion exercises to maintain FDP glide and prevent stiffness, while avoiding forceful gripping for the first few weeks. The surgical wound healed uneventfully. At six weeks, the patient had regained full flexion and extension of the finger without triggering and had returned to daily activities pain-free. At one year, he demonstrated a full range of motion, symmetric with the contralateral side, with no recurrence of triggering or paradoxical extension. Grip strength was comparable to that of the unaffected hand for his age. The overall outcome was excellent, with complete functional recovery and no complications. The patient and his parents were counselled on the rarity of the condition and advised to seek early review if new symptoms develop, although none have occurred to date.

## Discussion

Pathophysiology

Trigger finger in paediatric patients is believed to arise from a mismatch between the flexor tendon and the pulley mechanism, leading to binding of the tendon during movements [4]. In paediatric trigger thumbs, a nodular thickening (Notta’s nodule) on the flexor pollicis longus often causes locking at the A1 pulley. In PTFs (digits), the pathology can be more variable. Proposed mechanisms include a congenital thickening or anomaly of one slip of the flexor digitorum superficialis (which can snag and cause triggering) or tenosynovitis from underlying conditions leading to nodule formation. Our case, however, represents an unusual post-traumatic pathophysiology: a partial intratendinous rupture of the FDP tendon led to scar tissue and a flap of tendon projecting outward, which in turn caught on the A2 pulley. Essentially, the injury created an 'internal' tendon tear and nodule that functioned like a pulley-tendon mismatch. Because the tendon continuity was not grossly disrupted, this injury was not visible on ultrasound or MRI, yet it was enough to cause mechanical triggering of the finger.

It is important to distinguish an intratendinous rupture from other types of tendon injury. The term typically refers to a tear occurring within the substance of the tendon, often with an intact surrounding tendon sheath. In our patient, the tear was confined to the tendon’s interior (the outer tendon surface and sheath remained continuous). Literature definitions of intratendinous flexor tendon ruptures align with this scenario: a closed rupture within the tendon fibres, usually without an external laceration or sheath disruption. Such injuries are uncommon and often associated with underlying tendon degeneration or systemic disease in adults. Traumatic intratendinous ruptures in otherwise healthy tendons are quite rare. Our case underscores that even a partial, intratendinous tear can produce a palpable nodule of scar tissue that mechanically interferes with the pulley system.

Another key aspect of this case’s pathophysiology is the paradoxical extension of the DIP joint, known as the lumbrical plus phenomenon. Normally, the lumbrical muscle (which originates from the FDP tendon in the palm) assists with finger flexion by pulling on the extensor mechanism to extend the IP joints when the FDP is slack. However, if the FDP tendon is disrupted distal to the lumbrical origin, attempts at flexing the finger can instead transmit force through the intact lumbrical, causing the IP joints to extend, a paradoxical motion. This lumbrical plus deformity classically occurs after a complete FDP rupture or avulsion, where the proximal FDP stump retracts and tugs on the lumbrical. In our patient, the FDP was not completely ruptured; nevertheless, the partial tear and ensuing scar likely tethered the lumbrical enough to produce a mild lumbrical plus effect. Thus, even a minimally disrupted FDP can cause lumbrical tension if scar or partial fibre discontinuity alters the normal tendon-lumbrical relationship. During surgery, we noted the lumbrical was tight, and we released it to eliminate this effect. After lumbrical release and tendon repair, the paradoxical extension resolved, confirming the lumbrical’s role in the deformity. The lumbrical plus phenomenon is an important clinical sign; recognising it can point to an FDP lesion even when imaging is equivocal, as was the case in our patient.

Finally, it is worth differentiating the pathophysiology of an acute versus chronic presentation in traumatic trigger finger. In an acute phase, a frayed tendon tear might directly catch on a pulley edge, whereas in a delayed presentation (weeks to months post-injury), the primary issue is often a mass of fibrous scar tissue that developed at the injury site. Our patient presented about two months after trauma, enough time for significant scar and nodule formation, which explains why the triggering was due to a scar nodule under an intact pulley rather than an obvious loose tendon end. This insight aligns with the general understanding that intratendinous scar nodules can cause late-onset triggering after a partial tendon injury.

Clinical relevance and diagnosis

This case highlights several clinically relevant points for diagnosing unusual paediatric hand conditions. First, a thorough clinical examination was paramount. The presence of a palpable nodule on the tendon and the lumbrical plus sign (paradoxical DIP extension) were key clues pointing toward a tendon injury despite unremarkable imaging. In paediatric patients (and adults), a lumbrical plus deformity on exam should prompt suspicion of an FDP rupture or distal tendon anomaly. In our case, it was the examination findings and persistent symptoms that drove us to pursue surgical exploration despite normal ultrasound and MRI. Clinicians should be aware that high-resolution ultrasound and even MRI can sometimes miss a partial intratendinous tear, because the tendon may appear grossly continuous as it did here. Thus, when history (trauma), exam (triggering, paradoxical motion), and basic imaging do not align, one must consider proceeding to exploratory surgery rather than dismissing the symptoms.

Second, this case underlines the importance of a structured approach to case assessment. The timeline from injury to onset of triggering was crucial in understanding the mechanism (scar formation vs. acute catching). We found that about 6-8 weeks had elapsed, suggesting a scar-mediated triggering, which indeed was confirmed intraoperatively. Eliciting a clear history and timeline can guide the differential: an immediate trigger after injury might suggest acute tendon entrapment, whereas a delayed trigger suggests healing-related issues. We recommend that any paediatric finger trigger be evaluated for prior trauma, and if present, the interval noted.

Third, appropriate investigations should be utilised but interpreted with caution. Ultrasound is a useful first-line tool in hand injuries; in our patient, it correctly showed continuity of the flexor tendons, ruling out full-thickness rupture, but it was unable to diagnose the internal tear. MRI provided excellent anatomic detail and helped rule out pulley rupture or bony pathology, yet it too did not show the intratendinous split. It is possible that the tear was too small to discern or was obscured by surrounding tendon fibres. This reiterates that a normal MRI does not categorically exclude tendon pathology. In scenarios where clinical suspicion remains high, especially with a positive clinical sign like lumbrical plus, one should not be falsely reassured by negative imaging. In our practice, we advocate for exploration if a significant mechanical issue is evident clinically, after discussing risks with the patient and parents.

From a broader perspective, this case also reminds practitioners that PTF, particularly outside the thumb, is so rare that one must maintain a high index of suspicion for atypical causes. While adult trigger finger is usually idiopathic and easily managed, a child presenting with a trigger finger should prompt consideration of possible underlying causes such as trauma, metabolic disease, or anomaly. In our case, the trauma was evident, but in others, screening for conditions like mucopolysaccharidoses or rheumatoid arthritis may be warranted if multiple digits are involved. Basic laboratory tests or genetic tests might be indicated in such situations. In any paediatric trigger digit, one should examine all fingers and both hands, assess for hepatosplenomegaly or dysmorphic features (for storage diseases), and inquire about symptoms of systemic illness, to not miss an underlying diagnosis.

Literature context

We performed a literature review to contextualise our findings with previously reported cases of PTF, especially those related to trauma or tendon rupture. PTF was first distinguished from trigger thumb in older literature around the 1970s-1980s; since then, relatively few cases and series have been published due to its rarity. Cardon et al. reported on trigger fingers in children and emphasised that the condition is distinct from the more common paediatric trigger thumb, often requiring different treatment approaches [7]. Schaverien and Godwin performed a comprehensive review and noted that PTF often presents in children older than those with trigger thumb, frequently involves the long finger, and in many cases has no clear aetiology [8]. They also observed that, unlike trigger thumbs (which can sometimes be observed for spontaneous resolution), trigger fingers in children almost invariably ended up needing surgical release [8].

Traumatic cases

A systematic review by Wong et al. compiled 51 cases of PTF and noted that only 5% were post-traumatic in origin [5]. Those few trauma-related cases tended to involve older children (school-age), likely because significant finger trauma is uncommon in toddlers. The most frequently implicated trauma in literature has been physeal or bone injuries in the hand that secondarily cause tendon issues or direct tendon lacerations. For example, some reported cases involved fractures with excessive callus formation that led to tendon triggering, and others described partial lacerations that were initially missed. Seiler and Kerwin presented an interesting adolescent case of trigger finger caused by post-traumatic calcific tendinitis: a foreign-body reaction led to calcific deposit in the tendon over months, resulting in triggering [9]. In their case, the triggering developed seven months after the injury, illustrating a chronic inflammatory path. Our patient’s timeline was shorter, but also not immediate, fitting a pattern of delayed manifestation due to scar tissue proliferation rather than an acute mechanical blockade.

Flexor tendon ruptures in children are extremely uncommon without an open injury. Jackson et al. reported a case of closed partial FDP rupture in a paediatric patient with no history of trauma, and the child presented with the inability to flex the finger [10]. In that case, the rupture was apparently idiopathic and complete enough to cause loss of flexion. Essentially a 'silent' tendon rupture. Our case differs in that flexion was partially preserved, and the presentation was triggering rather than total loss of flexion. We believe ours is the first reported PTF due to a partial intratendinous tear with trauma.

Some adult literature helps illuminate our case as well. Yano et al. recently described an elderly patient with a degenerative partial rupture of FDP that presented as a soft tissue mass and locking of the finger [11]. In that case, like ours, imaging showed a mass and limited flexion; intraoperatively, an intratendinous split was found and simply resected. That patient had no trauma, but the mechanical problem, a lump within the tendon causing a block, is analogous. It reinforces that partial intratendinous ruptures can behave like space-occupying lesions in the tendon sheath.

Another adult case by Abe et al. demonstrated a lumbrical plus phenomenon due to repetitive trauma: the patient had paradoxical extension of the little finger from an adhesion between the FDP and lumbrical, and releasing the lumbrical resolved it [12]. This parallels our findings and supports our decision to release the lumbrical. In the paediatric realm, however, lumbrical plus is rarely documented; our case may be one of the few in a child. We hope that by reporting it, clinicians will remember to check for paradoxical extension in children with finger injuries. It can be easily overlooked unless specifically tested for: having the child make a fist while observing the DIP joints.

In summary, the literature indicates that PTF can arise from a variety of aetiologies: congenital anomalies, metabolic/inflammatory conditions, or, rarely, trauma. Our case enriches this context by providing a unique combination of factors (trauma + partial tendon rupture + lumbrical plus). It underlines the importance of considering tendon injury in the differential for paediatric triggering, especially after trauma, even if initial imaging is negative.

Case uniqueness

This case is unique in several respects. First, the cause of triggering was a closed intratendinous partial rupture of the FDP, a highly unusual lesion in a paediatric patient. Unlike the typical PTF cases, which involve thickened tendons or A1 pulley issues, our patient’s tendon had an internal tear that was not apparent externally. The tendon continuity and sheath were intact, making it a diagnostic challenge that defied non-invasive detection. Second, the case featured a paradoxical extension lumbrical plus deformity accompanying the trigger finger. While lumbrical plus deformity is known in adult tendon injuries, it is exceedingly rare to see it in a child, and even rarer for it to be caused by a partial tear rather than a complete rupture. The presence of this phenomenon in our patient added an extra layer of novelty and educational value, demonstrating that even a minimal tendon disruption can produce complex biomechanical effects. Third, this appears to be the first reported paediatric case linking direct trauma to a delayed-onset trigger finger via an intratendinous FDP injury. Prior reports of PTF have been idiopathic or post-traumatic, with different mechanisms such as calcific tendinitis or open injuries. Our case stands out as a clear instance where blunt trauma led to an internal tendon defect that subsequently caused triggering. The difficulty in diagnosis and the need for surgical confirmation further underscore its uniqueness.

Because of these features, our case contributes new insight into the 'spectrum' of PTF aetiologies. It reminds clinicians to think beyond the classic A1 pulley issue; rare entities like an intratendinous rupture can lurk beneath a seemingly simple trigger. The case also highlights the importance of intraoperative vigilance. We also illustrate that lumbrical release can be a useful adjunct in cases of lumbrical plus deformity, even if caused by a partial injury. More commonly, lumbrical plus is discussed in complete ruptures, but we found value in releasing it here to ensure no persistent paradoxical pull.

Management and treatment outcomes

The management of PTF is not standardised, owing to the condition’s rarity and variable causes. However, some general principles are agreed upon. If a PTF is mild and there is no underlying fixed contracture, a trial of conservative treatment is reasonable in very young patients. Conservative options include extension splinting of the affected finger or casting, especially in infants or toddlers, for a period to see if the triggering resolves. Unlike trigger thumbs, where observation often leads to spontaneous resolution in over 50% of cases by age 2, trigger fingers in children have a much lower rate of spontaneous improvement. In the systematic review by Shillingford et al., about 58% of PTFs treated non-operatively by splints or stretches showed resolution, whereas 87% of those treated with immediate surgery resolved [13]. Given the relatively lower success of non-operative management, many authors advocate for early surgery for PTFs, particularly if the child is older or if triggering is severe.

In our patient, conservative management was not indicated due to the traumatic aetiology and mechanical nature of the problem. Once it was clear that a structural lesion was present, albeit hidden on imaging, proceeding to surgical exploration was the appropriate choice. At surgery, our treatment strategy combined three steps: (1) release of the constricting pulley, (2) repair of the tendon, and (3) release of the lumbrical. This comprehensive approach addressed all aspects of the pathology.

Tendon repair in partial ruptures can be a debated issue. If a tendon is only slightly torn, some surgeons prefer to simply debride the frayed fibres and not place sutures, to avoid bulk and adhesion formation. An important guideline is the percentage of the tendon cross-section injured. Partial lacerations involving less than about 50% of the tendon often do not require suturing and can be managed by trimming the damaged fibres alone. Conversely, injuries involving more than 50%-60% of the tendon’s width are at risk of further tearing and may benefit from a suture repair to restore strength. In our patient, we opted to suture the rent because it created a segment that could continue to catch or extend if not secured. We used a 5-0 Prolene to minimise added bulk. Postoperatively, we allowed early motion, which is generally recommended after tendon repair to enhance healing and prevent adhesions.

Another management point was the lumbrical release. This is not a standard part of trigger finger surgery, but it became necessary due to the lumbrical plus deformity. By releasing the lumbrical, which was acting as an antagonist because of the tendon disruption, we eliminated the aberrant extension force. This step is more commonly described in the context of lumbrical plus finger correction for chronic FDP ruptures. In our case, we believe it contributed to the smooth post-op flexion. There is a slight loss of the lumbrical function (which assists in IP extension and MCP flexion), but the interossei largely compensate, and the patient did not notice any functional deficit from losing one lumbrical.

Our patient’s excellent outcome reflects the effectiveness of timely surgical intervention. At one year, he had a full range of motion and no pain or triggering; this is on par with outcomes reported in other PTF surgeries. In general, paediatric patients heal flexor tendon issues well and tend to regain motion if managed properly. Studies have shown high success rates for paediatric trigger digit releases in terms of resolving triggering [13]. Our case adds that even when the pathology is more complex, surgical repair can yield a full recovery. We acknowledge that close follow-up and therapy were important in our case to ensure a satisfactory result.

Lastly, our case exemplifies that a multidisciplinary approach, involving a plastic surgeon, radiologists, and hand therapists, is ideal in managing such cases. Radiology helped exclude other diagnoses; surgery addressed the problem; and hand therapy maximised the functional outcome. The lesson learned is to maintain a broad perspective: PTF is uncommon, but when encountered, one should evaluate thoroughly for unusual causes and tailor the treatment.

## Conclusions

We have presented a rare case of PTF caused by a closed intratendinous rupture of the FDP tendon following trauma. This case highlights the diagnostic challenge of intratendinous tendon injuries, which may be missed on standard imaging and require a high index of suspicion. Key points include the importance of a detailed clinical examination, particularly recognition of the lumbrical plus paradoxical extension sign, and a structured assessment with careful history, physical examination, and targeted imaging. Our surgical approach, involving pulley release, tendon repair, and lumbrical release, resulted in an excellent outcome with full restoration of function.

While PTF remains rare and lacks a clear consensus on management, this case demonstrates that when a definite mechanical problem is identified, especially after trauma, surgery can be both diagnostic and therapeutic. We suggest that intratendinous tendon rupture should be considered as a possible cause of PTF in patients with a relevant trauma history, even when initial imaging appears normal. This report adds to the limited literature on paediatric trigger digits by describing a novel mechanism of injury. Increased awareness of such atypical cases may aid in timely diagnosis and management. Continued reporting of rare presentations and outcomes will help establish clearer management guidelines. In conclusion, our patient’s successful recovery shows that a careful, individualised approach to rare paediatric hand injuries can achieve excellent results while contributing to our collective understanding of these uncommon conditions.
